# Farmacia en los sistemas de salud para la atención a pacientes ambulatorios: evolucionando

**DOI:** 10.23938/ASSN.1062

**Published:** 2023-12-26

**Authors:** Javier Garjón Parra, Lorea Sanz Álvarez

**Affiliations:** 1 Servicio Navarro de Salud-Osasunbidea Servicio Navarro de Salud-Osasunbidea (SNS-O) Subdirección de Farmacia y Prestaciones Servicio de Asesoría e Información del Medicamento Navarra Spain; 2 Instituto de Investigación Sanitaria de Navarra (IdiSNA) Navarra Spain

En los sistemas de salud nos enfrentamos a los retos de la polimedicación y de los riesgos de los medicamentos en una población envejecida. El aumento de las resistencias a antibióticos y el uso inadecuado de opioides y psicofármacos han puesto de manifiesto que el *primum non nocere* trasciende al paciente individual y afecta a la sociedad en su conjunto. Los farmacéuticos clínicos, integrados en servicios de farmacia de atención primaria y hospitalarios, estamos acometiendo estos problemas con estrategias como la conciliación y revisión de la medicación, la optimización de tratamientos y la desprescripción^1^^-^^3^. Todas ellas coinciden en implicar una valoración cuidadosa de los beneficios y riesgos de cada tratamiento. En sus últimos números, Anales del Sistema Sanitario de Navarra ha publicado varios artículos sobre distintos tipos de actuaciones para mejorar la farmacoterapia que van a servir para reflexionar sobre nuestra actividad y cómo puede evolucionar.

Un primer abordaje ha sido el basado en los medicamentos identificados como de alto riesgo que son aquellos que, cuando se utilizan incorrectamente, presentan una mayor probabilidad de causar daños graves a los pacientes^4^. Las intervenciones de los farmacéuticos clínicos y los enfoques multifacéticos han mostrado eficacia en reducir las prescripciones potencialmente inapropiadas^5^. Lo primero es tener bien caracterizados los riesgos de los fármacos. Para ello, Olry de Labry y col hacen una revisión paraguas (revisión de revisiones sistemáticas) sobre la seguridad de los antiinflamatorios no esteroideos (AINE) en pacientes con hipertensión, enfermedad cardiovascular, enfermedad renal crónica o cirrosis hepática que confirma la necesidad de evitarlos o, si se usan, hacerlo con mucha precaución^6^. También es preciso disponer de estrategias bien diseñadas. Así, Marquina-Márquez y col tratan el tema de la difícil desprescripción de benzodiacepinas realizando una investigación cualitativa en pacientes y médicos para identificar barreras y facilitadores. Concluyen con la importancia de promover intervenciones no farmacológicas, la formación en habilidades psicosociales y la promoción de equipos multidisciplinares^7^. Siguiendo este rumbo, en el Servicio Navarro de Salud-Osasunbidea (SNS-O) se han abordado por múltiples vías los riesgos de los AINE y la desprescripción de benzodiacepinas. Se han empleado boletines^8^^,^^9^, acciones formativas, alertas en la receta electrónica^10^ e información para pacientes^11^.

La validación farmacéutica de tratamientos con medicamentos de riesgo es una actuación que profundiza en este enfoque. En este número publicamos un estudio sobre las interacciones evitadas en los pacientes ambulatorios tratados con nirmatrelvir/ritonavir, medicamento para la COVID-19 que se caracteriza por interaccionar con numerosos fármacos^12^.

La continua incorporación de nuevos fármacos, algunos con un complejo perfil de seguridad o con incertidumbre sobre su balance entre beneficios, riesgos y costes, ha impulsado la atención farmacéutica directa para los pacientes crónicos ambulatorios a quienes se dispensa medicamentos en los servicios de farmacia hospitalaria. De esta forma se fomenta la adherencia al tratamiento, se gestionan los riesgos y también se monitoriza su efectividad. Es representativa la atención que se hace a pacientes con patologías reumáticas tratados con medicamentos biológicos o sintéticos dirigidos. Monforte Gasque y col evalúan en un estudio observacional retrospectivo en un hospital terciario, el grado de control del dolor y los analgésicos utilizados en pacientes con artritis reumatoide, artritis psoriásica o espondilitis anquilosante tratados con fármacos biológicos. Encuentran un 69% de los pacientes con dolor no controlado y severo, con un importante margen de mejora en el tratamiento del mismo^13^. También en pacientes con patologías reumáticas tratados con terapias biológicas, Caso-González y col comparan en un ensayo clínico una intervención farmacéutica basada en el modelo CMO (Capacidad, Motivación, Oportunidad) con la atención farmacéutica habitual. El número de pacientes es pequeño, a pesar de lo cual encuentran diferencias significativas a favor de la intervención estructurada en la adherencia a los tratamientos y en la experiencia de los pacientes y su satisfacción con los profesionales y servicios sanitarios^14^.

Todas estas intervenciones tienen en común que determinado medicamento es el criterio para acceder a ellas. Otro enfoque es que lo sea la situación del paciente. Se ha hecho mucho hincapié en los ancianos polimedicados, especialmente en las transiciones asistenciales por ser momentos de mayor riesgo. Marín-Gorricho y col estudian el impacto de la intervención farmacéutica en pacientes ancianos polimedicados hospitalizados en Geriatría. Se lograron significativas reducciones de prescripción potencialmente inadecuada y de problemas relacionados con los medicamentos^15^. Estos resultados concuerdan con los obtenidos en el SNS-O tras la integración de una farmacéutica en una unidad geriátrica de agudos^2^.

Entendemos que la integración de los servicios farmacéuticos en equipos asistenciales es el camino a seguir. Lo que es completamente aplicable a la atención primaria, desde el momento en que se está pasando de un enfoque más centrado en el medicamento y ligado a cambios en la farmacoterapia o transiciones asistenciales, a otro centrado en el paciente con una visión longitudinal, en la que es muy importante el elemento motivacional^16^. La optimización de la farmacoterapia no es algo separado de la atención sanitaria en general. No solo hay que tener en cuenta el medicamento y la enfermedad, sino además el entorno psicosocial del paciente. Por lo tanto, hay que mantener la continuidad de la atención e involucrar a pacientes y cuidadores^17^. Los y las profesionales de la medicina y de la enfermería deben implicarse, pero hay que ser conscientes que estas actuaciones consumen bastante tiempo de trabajo, con una escasez de personal sanitario que inevitablemente hará que deban centrarse cada vez más en las tareas que les son específicas. La respuesta a estas demandas pasa por la formación de equipos multidisciplinares en los que se aprovechan al máximo las habilidades de cada uno^18^. Todo esto se recoge en el modelo conceptual para el manejo de pacientes polimedicados que elaboraron Reeve y col^17^ mediante una síntesis de investigación cualitativa. Al profesional, presionado por la limitación de tiempo, le falta *espacio mental* para la evaluación de problemas complejos por lo que tiene dificultades para establecer objetivos terapéuticos y es reluctante a hacer cambios en la medicación. Por el contrario, con el trabajo en equipo se saca partido de las competencias de los compañeros. Se discuten los casos complejos, maximizando los conocimientos de cada uno, se comparte la carga de trabajo y el profesional se siente apoyado, resultando en una toma de decisiones sobre la prescripción con más confianza, con menos riesgos y asegurando la continuidad de la atención sanitaria^17^. Hay que admitir que la evidencia proveniente de investigación cuantitativa sobre la integración de farmacéuticos en equipos de atención primaria es escasa. Se han obtenido resultados de reducción de las prescripciones potencialmente inapropiadas y de la polimedicación, pero no en eventos clínicos o en mejoras de la calidad de vida^19^^,^^20^. Sin embargo, una evaluación independiente para el servicio nacional de salud inglés (NHS) concluyó que se habían obtenido mejoras en los conocimientos del equipo sobre medicamentos, en los flujos de trabajo, en la accesibilidad, y en la educación, seguridad y satisfacción de pacientes^21^. Esto condujo al NHS a incrementar la contratación de farmacéuticos clínicos en atención primaria^22^. Aunque la mayoría de estudios sobre el proceso de integración de farmacéuticos en los equipos de atención primaria proviene de países anglosajones, podemos extraer unas enseñanzas aplicables en nuestro medio. Para tener éxito, los farmacéuticos deben ganarse la confianza de médicos y pacientes, trabajando codo con codo con los demás profesionales sanitarios. La mejor manera de conseguirlo es estar en el mismo centro de salud. El modelo de servicios farmacéuticos debe ser flexible y adaptarse a las necesidades de los profesionales del equipo. Cuanto más conocen pacientes, médicos y enfermeros el trabajo de los farmacéuticos, más y mejor valoran su trabajo^22^^,^^23^.

Los farmacéuticos clínicos llevan tiempo informando sobre la eficiencia y la seguridad de la incorporación de nuevas terapias y de las propias estrategias de optimización de la farmacoterapia; tarea que, probablemente, se pueda hace de forma más certera trabajando dentro del propio equipo asistencial.

Podemos concluir que, si el trabajo en los servicios sanitarios se encamina a equipos multidisciplinares con tareas bien definidas, en colaboración y con intercambio de conocimientos, los farmacéuticos clínicos deben tener un papel muy relevante.
